# Safety and Effectiveness of Ustekinumab for Crohn’s Disease in Japanese Post-marketing Surveillance in Biologic-Naive and -Experienced Conriemed

**DOI:** 10.1093/crocol/otad001

**Published:** 2023-01-12

**Authors:** Katsumasa Nagano, Erina Hata, Teita Asano, Hiroaki Tsuchiya, Masayuki Takagishi, Hiroshi Yamazaki, Sonoko Tominaga, Takayuki Matsumoto

**Affiliations:** Janssen Pharmaceutical K.K., Medical affairs division, Tokyo, Japan; Janssen Pharmaceutical K.K., Medical affairs division, Tokyo, Japan; Janssen Pharmaceutical K.K., Medical affairs division, Tokyo, Japan; Janssen Pharmaceutical K.K., Medical affairs division, Tokyo, Japan; Janssen Pharmaceutical K.K., Research & Development division, Tokyo, Japan; Janssen Pharmaceutical K.K., Research & Development division, Tokyo, Japan; Janssen Pharmaceutical K.K., Research & Development division, Tokyo, Japan; Division of Gastroenterology, Department of Internal Medicine, School of Medicine, Iwate Medical University, Iwate, Japan

**Keywords:** ustekinumab, Crohn’s Disease Activity Index, bio-naive, bio-experienced

## Abstract

**Background:**

To present the real-world evidence on the safety and effectiveness of ustekinumab (UST) through 52-week treatment for Crohn’s disease (CD) under an analysis of post-market surveillance data in Japan.

**Methods:**

This prospective, post-marketing surveillance study was conducted in 341 patients from 91 medical facilities in Japan. Patients received UST 90 mg injected subcutaneously once every 12 weeks (or every 8 weeks if patients show weak effectiveness) after an induction dose given intravenously. Clinical response (100-point decrease in Crohn’s Disease Activity Index [CDAI] score), clinical remission (CDAI score of <150), steroid-free clinical remission, C-reactive protein, endoscopy, physician global assessment, and adverse drug reactions (ADRs) were evaluated through 52 weeks.

**Results:**

The overall rate of clinical remission was 49.2% at week 8 and 56.0% at week 52. The rate of clinical remission in biologic-naive patients was 75.9% and 66.7% at weeks 8 and 52, respectively, whereas the rate in biologic-experienced patients was 41.4% and 52.6% at weeks 8 and 52, respectively. For 52 weeks, the overall incidence of ADRs and serious adverse drug reactions (SADRs) was 11.7% and 6.7%, respectively. The most frequently reported SADRs was worsening of CD (1.8%). In multivariate analysis, ADRs incidence was significantly lower in patients with ileal involvement of CD (odds ratio = 0.25, 95% CI 0.07–0.85, *P* = .026), although disease location has no association with effectiveness of UST.

**Conclusions:**

The present study identified no new safety concerns and effectiveness for CD in Japanese patients treated with UST.

## Graphical Abstract

**Graphical Abstract:**
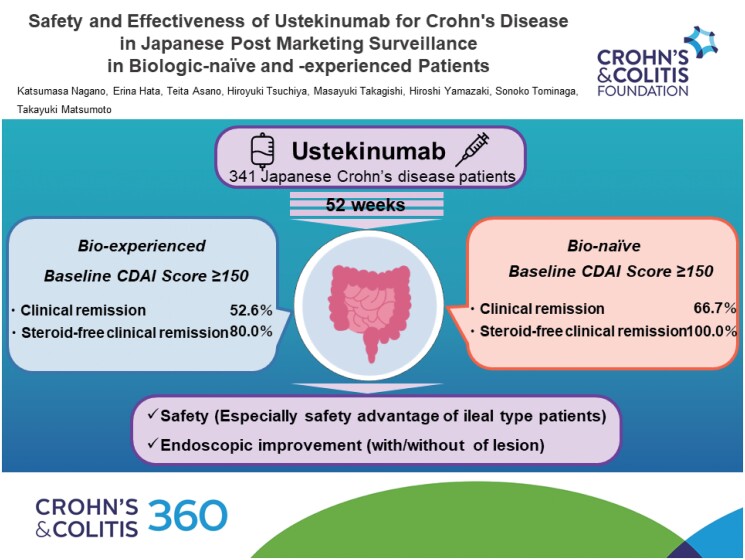

## Introduction

Crohn’s disease (CD) is an immune-mediated inflammatory disease of the gastrointestinal tract.^1^ Compared with Western countries, prevalence of CD is reported to be much lower in Asian countries, including Japan. However, recent epidemiological studies have suggested a rapidly increasing trend in the incidence of CD in Japan, probably due to the westernization of lifestyle and dietary habits.^2,3^ The annual prevalence of CD in Japan is 55.6 per 100.000 population.^4^ The primary treatment options for CD include glucocorticoids, immunosuppressants, tumor necrosis factor antagonists, or integrin inhibitors.^5,6^

The current treatment options for CD in Japan include 5-aminosalicylic acid (5-ASA), corticosteroids, immunomodulators (IM); azathioprine [AZA], 6-mercaptopurine [6-MP]), and biologics (infliximab [IFX], adalimumab [ADA], vedolizumab [VED], and ustekinumab [UST, Stelara]) for induction and maintenance.^7^ Biologics are effective for moderate-to-severe CD, but secondary loss of effectiveness and intolerance as well as serious adverse events (AEs) such as infection and malignancy have been reported.^8,9^

CD involves activation of Th1 or Th17 cells and the contribution of IL-12/23 as a pro-inflammatory cytokine to the condition.^10,11^ A genome-wide association study identified a significant association between CD and the IL-23 receptor.^12^ UST is a fully humanized immunoglobulin G1 kappa (IgG1κ) monoclonal antibody (mAb), which binds with high affinity to the p40 subunit common to both IL-12 and IL-23 and is approved for use in moderate-to-severe CD patients who were non-responders to previous treatment. Phase 2 (CERTIFI) and phase 3 clinical trials (UNITI1 and 2, IM-UNITI) have shown treatment with UST to induce and maintain remission in patients with CD.^6,13,14^

This post-marketing surveillance (PMS) study was performed to investigate the usage of UST in Japanese patients with CD. The dosage and administration approved in Japan are as follows: UST intravenous (IV) infusion, followed by subcutaneous (SC) administration of 90 mg at weeks 8 and 90 mg every 12 weeks thereafter. The dosing interval may be reduced to 8 weeks if response diminishes. Considering the differences in approval conditions and patient background with the United States and Europe, we believe that this study can report valuable treatment practices, and we will report the safety and efficacy of UST after 1-year induction and maintenance therapy in Japanese patients with moderate-to-severe CD.

## Materials and Methods

This study was a prospective, observational, and multicenter post-marketing study. The study was carried out according to the Japanese authorized standards for PMS and Good Post-marketing Study Practice without intervening in UST dosage and administration from May 24, 2017 to December 31, 2021. Good post-marketing study practice does not require the patients’ consent and approval of the study protocol by the institutional review board of each participating center. All authors accessed the study data and reviewed and approved the final manuscript.

### Study Population

In safety analysis, patients with moderate-to-severe active CD who had failed or were intolerant to earlier treatment initiated UST IV infusion. Patients with a history of the use of UST were excluded. In effective analysis, some patients who had Crohn’s Disease Activity Index (CDAI) scores <150 at baseline were also enrolled in this PMS, indicating that these patients were already in clinical remission before UST administration. We focused on the patients with CDAI scores ≥150 at baseline for the analysis evaluating the robustness of UST effectiveness.

### Patient Registration and Data Collection

Patients were enrolled on a central registration system in this prospective surveillance with a 1-year observation period. The investigator input information on patients included in this survey into the registration forms of the Electronic Data Capture (EDC) system and sent the information within 14 days after the first dosing date (counted as day 1) of UST IV infusion to the end of the observation period. All treatment decisions were taken at the discretion of the prescribing physician.

UST IV dosage (UST IV infusion 130 mg vial): UST was administered by IV infusion based on the patient’s weight (≤55 kg: 260 mg; >55 kg to ≤85 kg: 390 mg; and >85 kg: 520 mg). UST SC dosage (UST SC injection 45 mg syringe): UST 90 mg was injected SC 8 weeks after the first dosing of UST by IV infusion and thereafter SC injection of UST 90 mg was given once every 12 weeks. The dosing period may be shortened to once every 8 weeks if patients show weak effectiveness at the decision of the physician.

The observation period started from the first dosing date of UST IV infusion to week 52 or until treatment completion/discontinuation. Variables included patient characteristics: patient identification, past medical history, history of prior treatment (history of biological drug use), records of administration, history of prior therapies/concomitant therapies (drug therapies for CD, drug therapies for disease other than CD, therapies other than drug therapy), periodical examinations for tuberculosis or serious respiratory disease, effectiveness (CDAI, C-reactive protein [CRP], endoscopy, physician global assessment), safety, malignancy, laboratory tests, patient summary (patient outcomes), and patient status.

Treatment completion, discontinuation, suspension, and continuation and its reason were assessed in safety analysis set. The reasons of treatment discontinuation and suspension were divided into patient choice, AE, lack of effectiveness, transfer hospital, no visit, and other.

Safety evaluations included incidence of adverse drug reactions (ADRs), serious adverse drug reactions (SADRs), and factors affecting the safety of UST. Effectiveness evaluations included: (1) clinical response: 100-point decrease in CDAI score from the first UST administration until week 52 (when baseline CDAI score was ≥150); (2) clinical remission: as a CDAI score of <150 in patients prior to week 52 who had baseline CDAI score ≥150; (3) change in CRP from baseline to week 52; (4) endoscopy results: assessment of ileum and/or colon using endoscopy prior to week 52; and (5) factors associated with effectiveness at week 52 were investigated using the following subgroups: gender, age, previous use of biologics, recent previous use of biologics (IFX and ADA), previous use of steroids, concomitant use of steroids at baseline, concomitant use of AZA or 6-MP at baseline, perianal disease, extraintestinal manifestation (EIM), surgical history, patient hospitalization status, disease location, disease behavior, and baseline CDAI score.

### Statistical Analyses

Summary statistics of demographic and baseline characteristics were calculated for mean, SD, and median for continuous variables, and frequency and proportion for categorical variables. The frequencies of patients with ADRs and the incidence rates were tabulated by seriousness and preferred term (PT). CDAI is calculated from 8 items. If there were 4 or more measured items, the remaining 4 or less missing items were imputed by the last observation carried forward (LOCF) method in CDAI calculation. All other missing values were not imputed and excluded from the analysis as observed case approach.^15,16^ The summary statistics for CDAI and CRP values and changes at each visit, including baseline, were calculated. The clinical response and remission rates based on CDAI were calculated at each visit. The number of patients who achieved clinical remission at week 52 was tabulated. The number of patients with concomitant use of steroids and IM; patients with active or clinical remission by location at each visit were tabulated. Steroid-free remission was defined as steroid-free patients who were in clinical remission at each time point (weeks 8, 24, 36, and 52). Endoscopic assessment was calculated as the proportion of subjects with resolution of ulcer findings at the discretion of the investigator.

As an exploratory analysis, the odds ratio (OR) of the incidence of ADR or the presence of the clinical remission was compared between the levels of analysis factors, such as patient demographics and baseline characteristics. The 95% CI for each of the OR was calculated using univariate and multivariate logistic regression analysis with variable of patient backgrounds and baseline characteristics as covariates. The Wald test was applied for CI and the test for the OR. These tests were performed with the 2-sided significance level of 5%. The covariates with *P* value <.2 obtained from the univariate logistic regression analysis were used for multivariate logistic regression analysis.

### Ethical Considerations

This study was basically conducted in accordance with ethical principles originating from the Declaration of Helsinki. This study is not required for informed consent because of non-interventional study, but consent was obtained according to the site requirements. The protocol was approved by the Institutional Review Board according to the site requirements. The UMIN trial number is UMIN000043753.

## Results

### Patient Disposition

**Figure 1.**
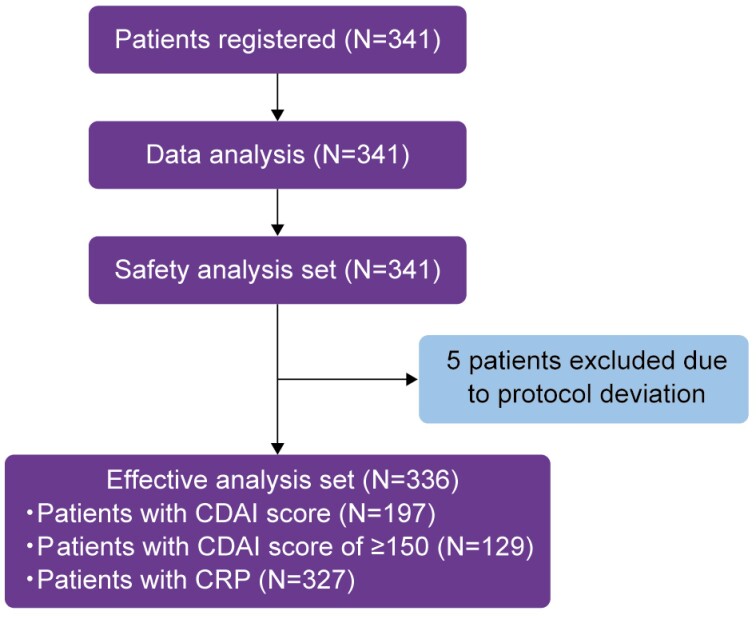
Patient flow diagram. Abbreviations: CDAI, Crohn’s Disease Activity Index; CRP, C-reactive protein; *N*, number.

A total of 341 patients were registered at 91 sites in this survey, and all were included in the safety analysis set. A total of 336 patients were included in the effectiveness analysis set, as 5 patients were excluded due to registration violations (Figure 1). Of the 341 patients, 274 (80.4%) patients continued UST treatment; 59 (17.3%) patients discontinued UST treatment (Supplementary Table S1), with the most common reasons being primary non-response (25 [42.4%] patients), transfer of health care system (12 [20.3%] patients), and AE (8 [13.6%] patients) (Supplementary Table S2); and 2 (0.6%) patients had suspended treatment (Supplementary Table S3). In the overall population, the number of patients for whom the dosing interval of 12 weeks was confirmed was 281, of whom 74 patients continued the dosing interval of 12 weeks and 207 patients had the dosing interval shortened to 8 weeks (Supplementary Table S4).

Overall, in safety analysis set, there was a higher proportion of males (230/341 [67.4%] patients) than females (111/341 [32.6%] patients). The median age was 38.0 years. The mean ± SD duration of CD at baseline was 11.0 ± 9.1 years with the majority of patients having ileocolonic (235/341 [68.9%] patients) and non-stricturing and non-penetrating (not including perianal fistula) 193 CD (179/341 [52.5%] patients). The most commonly used biologics and concomitant medications before UST treated were IFX (189/341 [76.8%] patients) and 5-ASA (243/341 [81.5%] patients), respectively. Patient demographics were analyzed comparatively for the safety analysis set and the effective analysis set. Since patients in remission with CDAI < 150 were included in full analysis set, thus patients with CDAI ≥150 were included in the effective analysis set (Table 1).

**Table 1.** Patient demographics and baseline disease characteristics.

Factor		Number of patients (%)			
		Safety analysis set		Effectiveness analysis set (CDAI score ≧150 at BL)	
Number of analysis set		341		129	
Gender	Male	230	(67.4)	93	(72.1)
	Female	111	(32.6)	36	(27.9)
Age (years)	<40	198	(58.1)	73	(56.6)
	≧40	143	(41.9)	56	(43.4)
	Mean ± SD	37.1 ± 13.4		38.0 ± 12.9	
	Median	38.0		38.0	
BMI (kg/m^2^)	<18.5	96	(28.2)	48	(37.2)
	≧18.5 to <25	191	(56.0)	71	(55.0)
	≧25 to <30	30	(8.8)	6	(4.7)
	≧30	8	(2.4)	4	(3.1)
	Mean ± SD	20.7 ± 3.8		20.1 ± 3.6	
	Median	20.2		19.7	
	Unknown	16	(4.7)	0	(0.0)
Duration of Crohn’s disease (years)	<5	93	(27.3)	36	(27.9)
	≧5 to <10	74	(21.7)	26	(20.2)
	≧10 to <15	56	(16.4)	24	(18.6)
	≧15	80	(23.5)	36	(27.9)
	Mean ± SD	11.0 ± 9.1		11.4 ± 9.2	
	Median	9.0		9.8	
	Unknown	38	(11.1)	7	(5.4)
Disease location	Ileal	68	(19.9)	19	(14.7)
	Colonic	40	(11.7)	15	(11.6)
	Ileocolonic	235	(68.9)	96	(74.4)
	Others	1	(0.3)	0	(0.0)
Disease behavior	Non-stricturing, non-penetrating	179	(52.5)	61	(47.3)
	Stricturing	152	(44.6)	65	(50.4)
	Penetrating	72	(21.1)	32	(24.8)
Smoking history	No	239	(70.1)	99	(76.7)
	Yes	63	(18.5)	18	(14.0)
	Current smoker	36	(57.1)	9	(50.0)
	Past smoker	27	(42.9)	9	(50.0)
	Undescribed	0	(0.0)	0	(0.0)
	Unknown	39	(11.4)	12	(9.3)
Perianal disease	No	163	(47.8)	85	(65.9)
	Yes	66	(19.4)	44	(34.1)
	Unknown	112	(32.8)	0	(0.0)
Comorbidities	No	222	(65.1)	77	(59.7)
	Yes	119	(34.9)	52	(40.3)
EIM	No	269	(78.9)	96	(74.4)
	Yes	72	(21.1)	33	(25.6)
Surgical history	No	188	(55.1)	63	(48.8)
	Yes	153	(44.9)	66	(51.2)
Patient hospitalization status (Inpatient/outpatient)	Outpatient	276	(80.9)	103	(79.8)
	Inpatient	65	(19.1)	26	(20.2)
Prior use of biologics	No	95	(27.9)	29	(22.5)
	Yes	246	(72.1)	100	(77.5)
By number of drugs	1	14	(5.7)	5	(5.0)
	≧2	232	(94.3)	95	(95.0)
Type of biologics used (There is duplication)	Infliximab	189	(76.8)	77	(77.0)
	Adalimumab	125	(50.8)	56	(56.0)
	Vedolizumab	1	(0.4)	1	(1.0)
	Others	1	(0.4)	0	(0.0)
Prior use of other treatment (Non-bio pharmacotherapy for Crohn’s disease) (There is duplication)	No	43	(12.6)	14	(10.9)
	Yes	298	(87.4)	115	(89.1)
	Steroid	119	(39.9)	58	(50.4)
	AZA	73	(24.5)	25	(21.7)
	6-MP	22	(7.4)	9	(7.8)
	Methotrexate	2	(0.7)	2	(1.7)
	5-ASA	243	(81.5)	95	(82.6)
	Antibiotics	23	(7.7)	12	(10.4)
	Enteral nutrition	31	(10.4)	14	(12.2)
	Others	76	(25.5)	41	(35.7)
Concomitant medication at baseline (For Crohn’s disease) (There is duplication)	No	54	(15.8)	20	(15.5)
	Yes	287	(84.2)	109	(84.5)
	Steroid	97	(33.8)	49	(45.0)
	AZA	68	(23.9)	23	(21.1)
	6-MP	21	(7.3)	8	(7.3)
	Methotrexate	2	(0.7)	2	(1.8)
	5-ASA	239	(83.3)	91	(83.5)
	Antibiotics	11	(3.8)	6	(5.5)
	Enteral nutrition	31	(10.8)	12	(11.0)
	Others	75	(26.1)	40	(36.7)
Baseline CDAI score	No	141	(41.3)	0	(0.0)
	Yes	200	(58.6)	129	(100.0)
	<150	69	(34.5)	0	(0.0)
	≧150 to <220	56	(28.0)	54	(41.9)
	≧220 to <450	72	(36.0)	72	(55.8)
	≧450	3	(1.5)	3	(2.3)

Abbreviations: 5-ASA, 5-aminosalicylic acid; 6-MP, 6-mercaptopurine; AZA, azathioprinee; BMI, body mass index; CDAI, Crohn’s Disease Activity Index; EIM, extraintestinal manifestation.

### Effectiveness

#### Clinical findings

**Figure 2.**
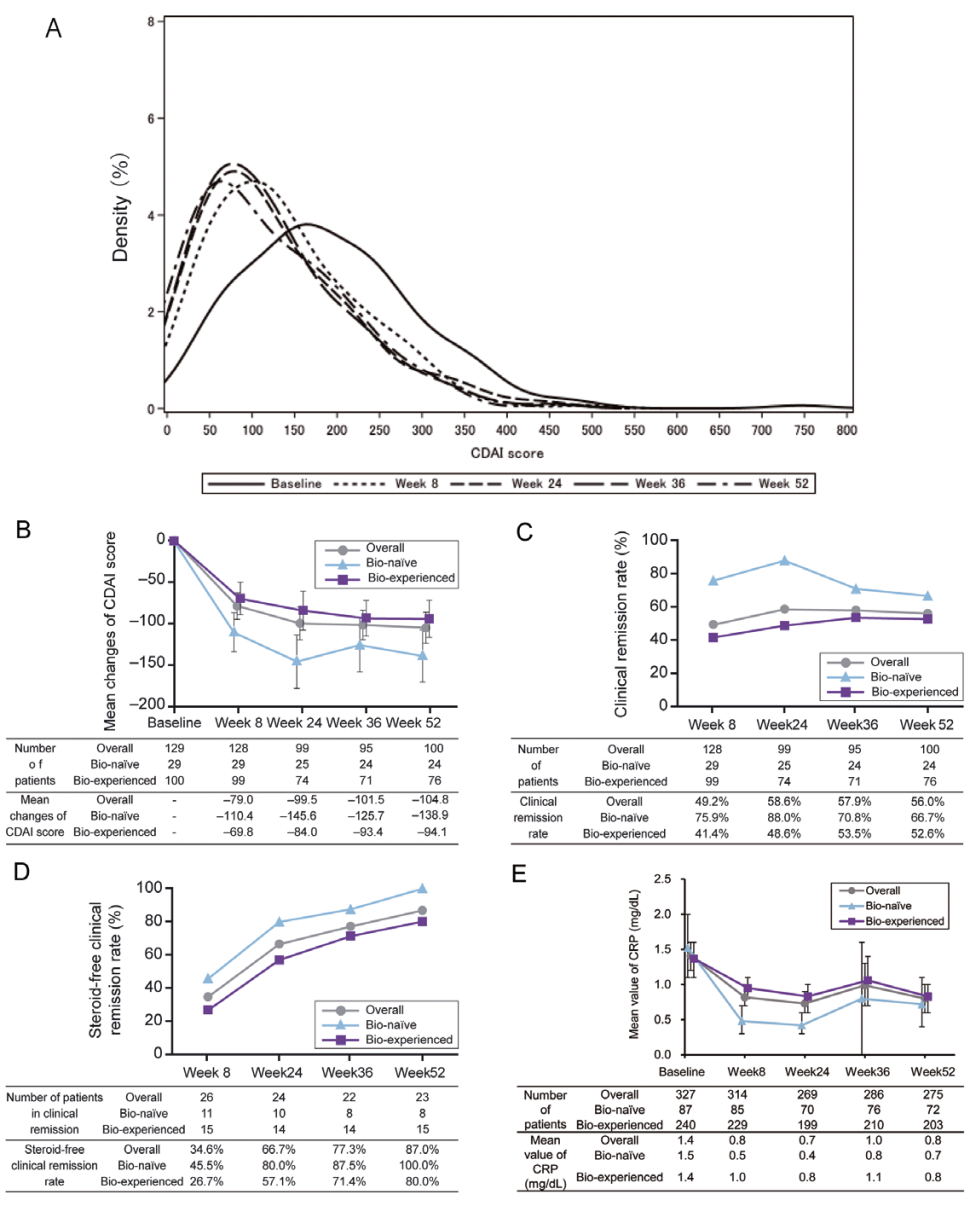
Effectiveness of ustekinumab from baseline to week 52. A, Density with overall patients in effective analysis set (*N* = 336) distribution of CDAI scores was shown at each visit. B, CDAI changes from baseline from the patients who had CDAI score of equal or more than 150 at baseline. C, Clinical remission rate in all patients (overall), Bio-naive, and Bio-experienced populations who had CDAI score of equal or more than 150 at baseline. D, Steroid-free clinical remission rate of patients who had CDAI score of equal or more than 150 at baseline. Steroid-free clinical remission; achievement of clinical remission and corticosteroids withdrawal in the patients who were receiving corticosteroids at baseline. E, Mean value of CRP in CRP analysis set (*N* = 327) was shown. Error bar shows 95% CIs. Abbreviations: biologic-experienced, Bio-experienced; biologic-naive, Bio-naive; CDAI, Crohn’s Disease Activity Index; CRP, C-reactive protein; *N*, number.

**Figure 3.**
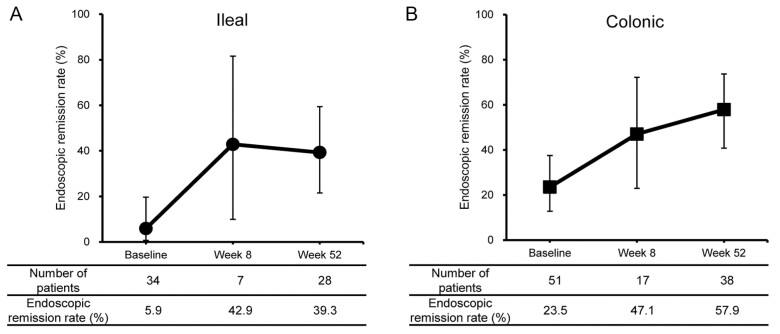
Proportion of patients with no endoscopic lesion site of disease. In effective analysis sets (*N* = 336), endoscopy was performed as needed. Endoscopic evaluation (active or remission) was conducted at the discretion of physicians. A, Percentage of patients with lesions in the ileal location. B, Percentage of patients with lesions in the colonic location. Abbreviation: *N*, number.

**Figure 4.**
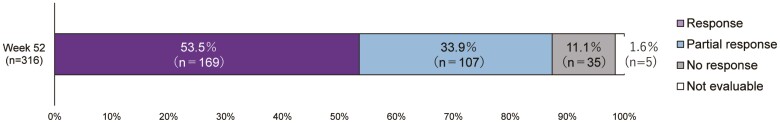
Physician’s global assessment. Physician’s global assessment (response/partial response/no response) was evaluated at week 52. Abbreviation: *N*, number.

The overall CDAI scores decreased in a time-dependent manner through week 52 (Figure 2A and B). The decrease in mean CDAI score was higher in biologic (Bio)-naive patients compared with Bio-experienced patients (Supplementary Figure S1A and B). To evaluate the effectiveness of UST, an analysis was performed in a group in which patients with CDAI < 150 at baseline were excluded from the evaluation of clinical remission and clinical response. The clinical remission rate (CDAI <150) at week 8 was 49.2%, increased to 56.0% at week 52. The clinical remission rates and response rates in bio-naive patients were greater than that in Bio-experienced patients at all time points (Figure 2C and Supplementary Figure S2). Both groups improved their clinical remission rates from baseline, but the rate at week 52 tended to be higher in the Bio-naive group. The proportion of steroid-free patients who were in clinical remission (steroid-free clinical remission rate) at each time point was higher in Bio-naive patients (100% at week 52) as compared with Bio-experienced patients (80% at week 52) (Figure 2D). CRP levels decreased from week 8 and continued to decrease through week 52 (Figure 2E). Finally, endoscopy was performed for the assessment of mucosal healing. The proportion of patients who had ileum and colonic endoscopic remission increased at week 52 compared with baseline following administration of UST (Figure 3). At week 52 based on physician global assessment, 169/316 patients (53.5%) had a response, 107 patients (33.9%) had partial response whereas 35 patients (11.1%) had no response (Figure 4).

#### Factors affecting the effectiveness of UST for clinical remission

When focused on the patients with CDAI ≥150 at baseline, previous use of biologics (OR 0.32, CI 0.13–0.80, *P* = .015), concomitant use of IM at baseline (OR 0.34, CI 0.15–0.80, *P* = .014), a surgical history for CD (OR 0.27, CI 0.13–0.55, *P* < .001), and higher baseline CDAI scores (OR 0.99, CI 0.98–0.99, *P* < .001) negatively affected clinical remission in logistic regression univariate analysis. In this study, among UST-treated patients, the patients with non-stricturing, and non-penetrating disease had more effectiveness as compared with patients who had developed stricturing and penetration complications (OR 2.22, CI 1.10–4.50, *P* = .027). Multivariate analysis showed that clinical remission was lower in patients having high baseline CDAI score (OR 0.99, CI 0.98–1.00, *P* < .001) (Table 2).

**Table 2.** Factors affecting the effectiveness of ustekinumab identified by multivariate logistic regression analysis for remission.

Factor		Number of patients (%)		Number of patients with remission (%)	Univariate			Multivariate			
					OR	95% CI	*P*	OR	95% CI		*P*
Number of effectiveness analysis set		129		67	(51.9)	—	—	—	—	—	—
Gender	Male	93	(72.1)	46	(49.5)	—	—	—	—	—	—
	Female	36	(27.9)	21	(58.3)	1.43	0.66, 3.11	.367	—	—	—
Age	Years (continuous)	129	(100.0)	—	—	0.97	0.95, 1.00	.054	0.98	0.95, 1.02	.356
Previous use of biologics	No	29	(22.5)	21	(72.4)	—	—	—	—	—	—
	Yes	100	(77.5)	46	(46.0)	0.32	0.13, 0.80	.015*	0.61	0.19, 1.94	.401
Recent previous use of infliximab	No	77	(59.7)	41	(53.2)	—	—	—	—	—	—
	Yes	52	(40.3)	26	(50.0)	0.88	0.43, 1.78	.717	—	—	—
Recent previous use of adalimumab	No	81	(62.8)	47	(58.0)	—	—	—	—	—	—
	Yes	48	(37.2)	20	(41.7)	0.52	0.25, 1.07	.074	0.76	0.30, 1.91	.565
Previous use of steroids	No	71	(55.0)	39	(54.9)	—	—	—	—	—	—
	Yes	58	(45.0)	28	(48.3)	0.77	0.38, 1.54	.452	—	—	—
Concomitant use of steroids at BL	No	80	(62.0)	40	(50.0)	—	—	—	—	—	—
	Yes	49	(38.0)	27	(55.1)	1.23	0.60, 2.50	.574	—	—	—
Concomitant use of AZA or 6-MP at BL	No	98	(76.0)	57	(58.2)	—	—	—	—	—	—
	Yes	31	(24.0)	10	(32.3)	0.34	0.15, 0.80	.014*	0.37	0.13, 1.02	.055
Perianal disease	No	85	(65.9)	40	(47.1)	—	—	—	—	—	—
	Yes	44	(34.1)	27	(61.4)	1.79	0.85, 3.75	.125	1.26	0.51, 3.10	.613
Comorbidities	No	77	(59.6)	41	(53.2)	—	—	—	—	—	—
	Yes	52	(40.3)	26	(50.0)	0.88	0.43, 1.78	.717	—	—	—
EIM	No	96	(74.4)	48	(50.0)	—	—	—	—	—	—
	Yes	33	(25.6)	19	(57.6)	1.36	0.61, 3.01	.453	—	—	—
Surgical history	No	63	(48.8)	43	(68.3)	—	—	—	—	—	—
	Yes	66	(51.2)	24	(36.4)	0.27	0.13, 0.55	<.001*	0.48	0.20, 1.19	.115
Patient hospitalization status	Outpatient	103	(79.8)	52	(50.5)	—	—	—	—	—	—
	Inpatient	26	(20.2)	15	(57.7)	1.34	0.56, 3.19	.512	—	—	—
L1	No	110	(85.3)	58	(52.7)	—	—	—	—	—	—
	Yes	19	(14.7)	9	(47.4)	0.81	0.30, 2.14	.666	—	—	—
L2	No	114	(88.4)	57	(50.0)	—	—	—	—	—	—
	Yes	15	(11.6)	10	(66.7)	2.00	0.64, 6.22	.231	—	—	—
L3	No	33	(25.6)	19	(57.6)	—	—	—	—	—	—
	Yes	96	(74.4)	48	(50.0)	0.74	0.33, 1.64	.453	—	—	—
B1	No	68	(52.7)	29	(42.6)	—	—	—	—	—	—
	Yes	61	(47.3)	38	(62.3)	2.22	1.10, 4.50	.027*	1.33	0.43, 4.17	.622
B2	No	64	(49.6)	38	(59.4)	—	—	—	—	—	—
	Yes	65	(50.4)	29	(44.6)	0.55	0.27, 1.11	.095	0.83	0.29, 2.36	.720
B3	No	97	(75.2)	54	(55.7)	—	—	—	—	—	—
	Yes	32	(24.8)	13	(40.6)	0.54	0.24, 1.23	.142	1.18	0.39, 3.58	.774
Baseline CDAI score	Score (continuous)	129	(100.0)	—	—	0.99	0.98, 0.99	<.001*	0.99	0.98, 1.00	<.001*

Abbreviations: 6-MP, 6-mercaptopurine; AZA, azathioprine; B1, behavior (non-stricturing, non-penetrating); B2, behavior (stricturing); B3, behavior (penetrating); BL, baseline; CDAI, Crohn’s Disease Activity Index; EIM, extraintestinal manifestation; L1, ileal; L2, colonic; L3, ileocolonic; OR, odds ratio. The factors of *P* < .2 in univariate analysis are used for multivariate analysis.

*The test result was given at *P* < .050.

### Safety

During 52 weeks, the overall incidence of ADRs and SADRs was 11.7% and 6.7%, respectively. The incidence of ADRs by MedDRA PT was highest for worsening of CD (1.8%), followed by pyrexia (1.2%), anal abscess, and upper respiratory tract inflammation (0.9% each). The most frequently reported SADR was worsening of CD (1.8%) (Table 3). In logistic regression multivariate analysis, the ADR incidence was significantly higher in patients with comorbidities (OR 2.36, 95% CI 1.16–4.78, *P* = .017) and significantly lower incidences of ADRs in patients with ileal involvement of CD (OR 0.25, CI 0.07–0.85, *P* = .026). Other negative factors affecting the safety of UST identified by the ORs for ADRs were prior biologic use, surgical history, and patient hospitalization status (Table 4). Worsening of CD was not observed in ileal involvement of CD patients who reported ADRs. In this study, there was no notable association in the incidence of ADRs between patients by prior use of biologics, prior use of steroids, concomitant use of steroids at baseline, concomitant use of IM (AZA or 6-MP), EIM, hospitalization status, or disease behavior.

**Table 3.** Incidence of adverse drug reactions.

*N* = 341	ADR	SADR	Non-SADR
Number of patients	40	23	21
Number of events	57	27	30
Incident rate (%)	11.7%	6.7%	6.2%
Common ADRs observed in >0.5% patients, *n* (%)			
Worsening of CD	6 (1.8%)	6 (1.8%)	0 (0.0%)
Pyrexia	4 (1.2%)	0 (0.0%)	4 (1.2%)
Anal abscess	3 (0.9%)	3 (0.9%)	0 (0.0%)
Upper respiratory tract inflammation	3 (0.9%)	0 (0.0%)	3 (0.9%)
Malaise	2 (0.6%)	0 (0.0%)	2 (0.6%)
Influenza	2 (0.6%)	0 (0.0%)	2 (0.6%)
Nasopharyngitis	2 (0.6%)	0 (0.0%)	2 (0.6%)
Headache	2 (0.6%)	0 (0.0%)	2 (0.6%)
Intestinal obstruction	2 (0.6%)	2 (0.6%)	0 (0.0%)

Abbreviations: ADR, adverse drug reaction; CD, Crohn’s disease; SADR, serious adverse drug reaction. ADR occurred more than 0.5% is shown.

**Table 4.** Factors affecting the safety of ustekinumab identified by odds ratio for adverse drug reactions.

Factor		Number of patients (%)		Number of patients with ADR (%)		Univariate			Multivariate		
						OR	95% CI	*P*	OR	95% CI	*P*
Number of safety analysis set		341		40	(11.7)	—	—	—	—	—	—
Gender	Male	230	(67.4)	28	(12.2)	—	—	—	—	—	—
	Female	111	(32.6)	12	(10.8)	0.87	0.43, 1.79	.714	—	—	—
Age	Years (continuous)	341	(100.0)	—	—	0.99	0.96, 1.01	.327	—	—	—
Previous use of biologics	No	95	(27.9)	7	(7.4)	—	—	—	—	—	—
	Yes	246	(72.1)	33	(13.4)	1.95	0.83, 4.57	.125	1.38	0.56, 3.39	.477
Recent previous use of infliximab	No	194	(56.9)	19	(9.8)	—	—	—	—	—	—
	Yes	147	(43.1)	21	(14.3)	1.54	0.79, 2.97	.204	—	—	—
Recent previous use of adalimumab	No	242	(71.0)	28	(11.6)	—	—	—	—	—	—
	Yes	99	(29.0)	12	(12.1)	1.05	0.51, 2.17	.886	—	—	—
Previous use of steroids	No	222	(65.1)	24	(10.8)	—	—	—	—	—	—
	Yes	119	(34.9)	16	(13.4)	1.28	0.65, 2.52	.472	—	—	—
Concomitant use of steroids at BL	No	244	(71.6)	29	(11.9)	—	—	—	—	—	—
	Yes	97	(28.4)	11	(11.3)	0.95	0.45, 1.98	.888	—	—	—
Concomitant use of AZA or 6-MP at BL	No	252	(73.9)	28	(11.1)	—	—	—	—	—	—
	Yes	89	(26.1)	12	(13.5)	1.25	0.60, 2.57	.550	—	—	—
Perianal disease	No	163	(47.8)	25	(15.3)	—	—	—	—	—	—
	Yes	66	(19.4)	6	(9.1)	0.71	0.28, 1.77	.460	—	—	—
	Unknown	112	(32.8)	9	(8.0)						
Comorbidities	No	222	(65.1)	17	(7.7)	—	—	—	—	—	—
	Yes	119	(34.9)	23	(19.3)	2.89	1.48, 5.66	.002*	2.36	1.16, 4.78	.017*
EIM	No	269	(78.9)	31	(11.5)	—	—	—	—	—	—
	Yes	72	(21.1)	9	(12.5)	1.10	0.50, 2.42	.819	—	—	—
Surgical history	No	188	(55.1)	15	(8.0)	—	—	—	—	—	—
	Yes	153	(44.9)	25	(16.3)	2.25	1.14, 4.44	.019*	1.99	0.97, 4.10	.061
Patient hospitalization status	Outpatient	276	(80.9)	29	(10.5)	—	—	—	—	—	—
	Inpatient	65	(19.1)	11	(16.9)	1.73	0.82, 3.69	.152	1.64	0.74, 3.63	.220
L1	No	273	(80.1)	37	(13.6)	—	—	—	—	—	—
	Yes	68	(19.9)	3	(4.4)	0.29	0.09, 0.99	.047*	0.25	0.07, 0.85	.026*
L2	No	301	(88.3)	33	(11.0)	—	—	—	—	—	—
	Yes	40	(11.7)	7	(17.5)	1.72	0.71, 4.20	.232	—	—	—
L3	No	106	(31.1)	10	(9.4)	—	—	—	—	—	—
	Yes	235	(68.9)	30	(12.8)	1.40	0.66, 2.99	.378	—	—	—
B1	No	162	(47.5)	20	(12.3)	—	—	—	—	—	—
	Yes	179	(52.5)	20	(11.2)	0.89	0.46, 1.73	.737	—	—	—
B2	No	189	(55.4)	21	(11.1)	—	—	—	—	—	—
	Yes	152	(44.6)	19	(12.5)	1.14	0.59, 2.21	.692	—	—	—
B3	No	269	(78.9)	30	(11.2)	—	—	—	—	—	—
	Yes	72	(21.1)	10	(13.9)	1.29	0.60, 2.77	.522	—	—	—

Abbreviations: 6-MP, 6-mercaptopurine; ADR, adverse drug reaction; AZA, azathioprine; B1, behavior (non-stricturing, non-penetrating); B2, behavior (stricturing); B3, behavior (penetrating); BL, baseline; EIM, extraintestinal manifestation; L1, ileal; L2, colonic; L3, ileocolonic; OR, odds ratio. The factors of *P* < .2 in univariate analysis are used for multivariate analysis.

*The test result was given at *P* < .050.

## Discussion

This PMS presents safety and effectiveness data following 1-year treatment with an antibody to IL12/IL23 (UST) in Japanese patients with moderate-to-severe CD. Results showed that the effectiveness response of UST was observed as early as week 8 and was maintained until week 52 with no new safety concerns.

The overall CDAI score decreased in a time-dependent manner from baseline through week 52. Higher fall in CDAI was observed in Bio-naive patients compared with those who had previous history of biologics. This observation is in line with findings of the modelling study which reported UST as the first-line biologic therapy yields greater quality adjusted life years in moderate-to-severe CD patients at the end of 1 year.^17^ The clinical remission rate in the present study are higher than that reported by a retrospective study from Japan, which was 27% and 32.4% at weeks 8 and 52, respectively.^18^ The findings from the present study also confirm that in patients who were responders in the UST induction phase (at week 8), clinical remission and response rates were maintained through week 52 of the maintenance period in both Bio-naive and -experienced, with higher response and remission rates seen in the Bio-naive population. These findings are consistent with other long-term studies as well as a nationwide prospective observational cohort study with UST.^13,19^ Considering the transition of clinical remission rate through 52 weeks, the peak in Bio-experienced patients was at week 36 (53.5%), while the peak of clinical remission rate in bio-naive patients was at week 24 (88.0%). Although the persistence of UST was comparable between Bio-naive (82.1%) and Bio-experienced patients (79.7%), a difference in onset of response to UST therapy was observed between the Bio-naive and the Bio-experienced populations.

In the steroid-free clinical remission rate, the majority of patients who were receiving corticosteroids at baseline were able to withdraw corticosteroids successfully by week 52. A retrospective study assessing the actual effectiveness and safety of UST in second- or third-line CD in a large cohort in Italy and IM-UNITI study suggests that UST effects in CD may be steroid independent.^13,20^ These results suggest that UST may enable steroid withdrawal in the treatment of CD and reduce the use and burden of medications. On the other hand, there are controversial views on the concomitant use of IM with UST in CD. A systematic review and meta-analysis have reported the concomitant use of an IM with UST to be more effective than UST monotherapy for induction therapy.^21^ In contrast, the present study found the concomitant use of IM at baseline to negatively affect clinical remission at week 52. Similarly, data from the IM-UNITI LTE study and another retrospective cohort study had reported that the concurrent use of IM does not increase remission effectiveness.^18,22^ However, the results obtained with this study suggest that IM-treated subjects may have more severe disease than non-treated subjects, and the relationship between UST and IM combination needs to be further investigated.

In the present study, endoscopic improvement was observed in both ileum and colon. In addition, the present study also found lower ADR in patients with ileal involvement of CD following 52 weeks of treatment. Considering that exacerbation of CD was the most common ADR among ADRs, there was no significant difference in effectiveness by disease location. However, there is a possibility that UST exerted a positive effect on ileal lesions. Results from ENEIDA registry study have reported ileal disease location to be associated with better response to treatment with UST in the short term.^23^ The present study findings assume significance in the face of the suggestion for a new classification for CD as ­either ileum-dominant or isolated colonic disease.^24^ This observation may suggest that patients with ileitis are prone to less ADR of UST, while further evidence is needed to make a conclusion.

The safety profile of UST treatment in CD has been established previously. In a multicenter trial in Israel and a retrospective UST cohort study in Scotland, the incidence of any AE was low with UST treatment.^25,26^ The IM-UNITI Trial reported similar rates of AE between UST treated and placebo group following 5 years of treatment.^27^ Another retrospective study evaluating the real-world effectiveness associated with 52-week UST treatment in Japanese population has also reported the AEs to be mild and low in frequency (9.5%).^18^ In the present PMS, the frequency of ADRs and SADRs was generally low following a UST maintenance treatment of 52 weeks. No new safety signals were identified as compared with the findings from the interim analysis at week 8.^28^

There are several limitations in this study. Firstly, the present PMS being registered study has missing values for CDAI and CRP. This is one of the main limitations. The use of LOCF compliments the missing values in the study. Also, the use of observed case analysis for CDAI scores usually provides a better percentage of efficacy than intent to treat analysis. Clinical response, clinical remission, and steroid-free clinical remission were assessed using CDAI score evaluated by observed analysis. They could have been the reason for a 100% steroid-clinical remission shown in the present study. Secondly, whereas the data were compared between Bio-naive and -experienced groups, there was no placebo control group. Lastly, only Japanese patients were included in the present study.

In conclusion, no new concerns of safety or effectiveness were identified following UST therapy in Japanese patients with moderate-to-severe CD. We found the use of UST to be more effective in Bio-naive patients as compared with those who had prior exposure to biologics and most of patients who achieved clinical remission was steroid free at week 52. Though there is no clear difference in effectiveness among disease location, the ADR incidence was significantly less frequent in CD patients who had ileal disease location compared with CD patients with ileocolonic or colonic disease location.

## Supplementary Material

otad001_suppl_Supplementary_Material

otad001_suppl_Supplementary_Table

## Data Availability

The data underlying this article cannot be shared publicly due to confidentiality clauses signed with participating medical institutions.
