# Supplementary material for: Safety and Effectiveness of Ustekinumab for Crohn’s Disease in Japanese Post-marketing Surveillance in Biologic-Naive and -Experienced Conriemed
Source: Crohns Colitis 360. 2023 Jan 12;5(1):otad001. doi: 10.1093/crocol/otad001 (PMC9912369; doi:10.1093/crocol/otad001)

**Supplemental Figure1.** **Density with distribution of CDAI scores from baseline to week 52 in subgroups according to the status of previous biologic use**

197 patients had CDAI scores recorded at baseline and at least once during the study period. Among them, density with biologic (Bio)-naïve (N=45) (A) and Bio-experienced patients (N=152) (B) distribution of CDAI scores were shown at each visit. CDAI, Crohn’s Disease Activity Index; N, Number.


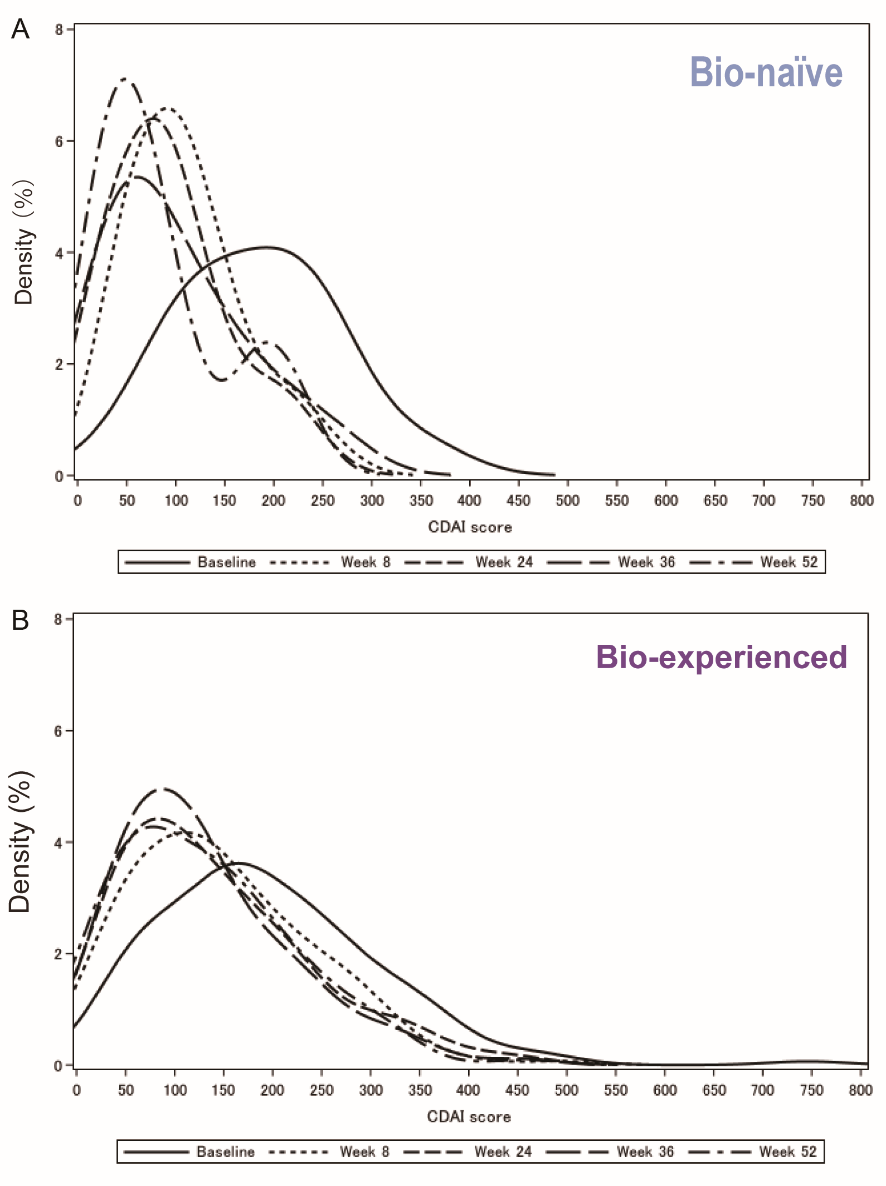


**Supplemental Figure 2. Clinical response rate**

Clinical response rate in all patients (overall), biologic (Bio)-naïve, and Bio-experienced populations who had CDAI score of equal or more than 150 at baseline. CDAI decreased by 100 or more from baseline (when baseline CDAI is 220 or more and 248 or less, when CDAI reaches less than 150). CDAI, Crohn’s Disease Activity Index.


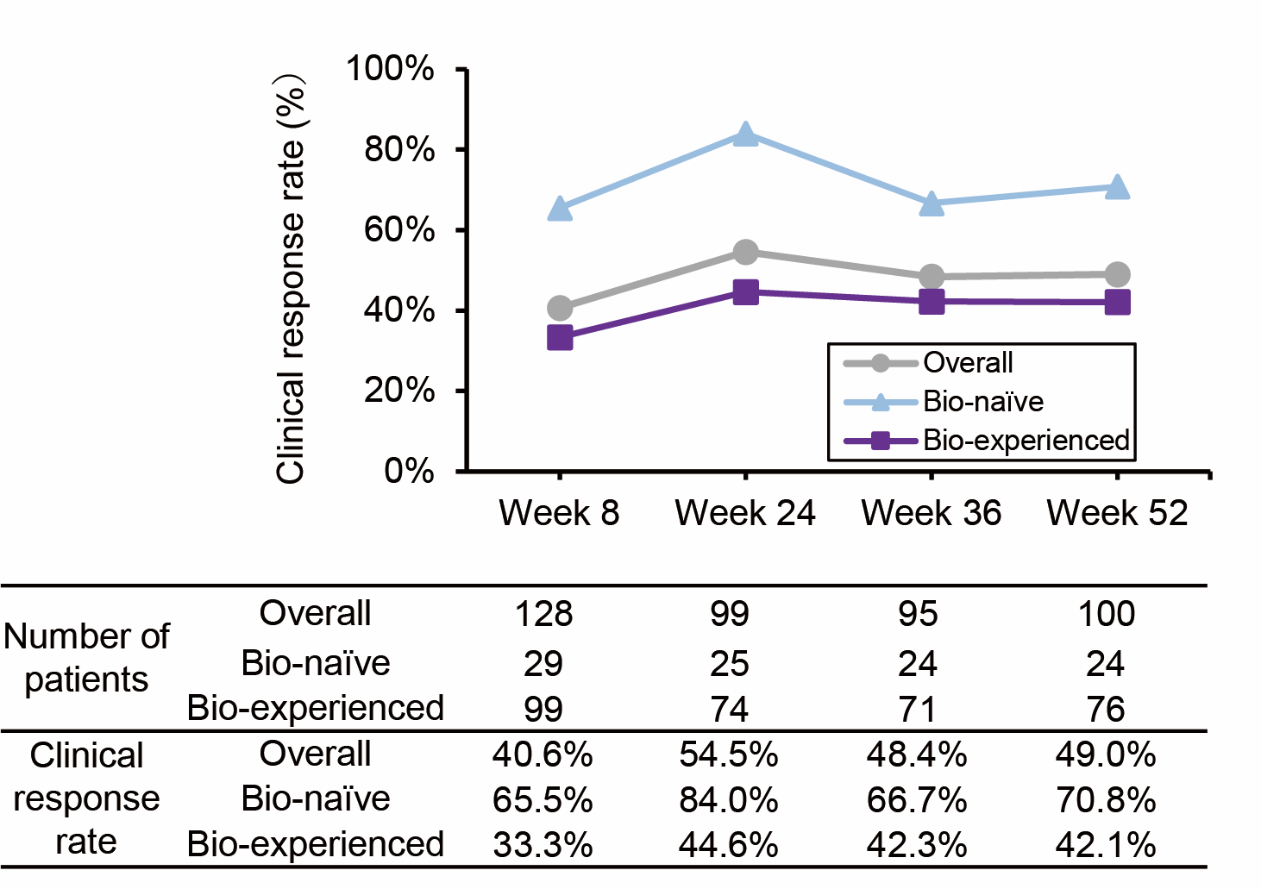

Supplement: otad001_suppl_Supplementary_Material [file otad001_suppl_supplementary_material.docx]
