# Supplementary material for: Safety and Effectiveness of Ustekinumab for Crohn’s Disease in Japanese Post-marketing Surveillance in Biologic-Naive and -Experienced Conriemed
Source: Crohns Colitis 360. 2023 Jan 12;5(1):otad001. doi: 10.1093/crocol/otad001 (PMC9912369; doi:10.1093/crocol/otad001)
Supplement: otad001_suppl_Supplementary_Table [file otad001_suppl_supplementary_table.docx]

# SUPPLEMENTAL MATERIAL

## Supplemental Table 1 Number of Treatment Discontinuation and Suspension

|  | **Number of patients (%)** | **Number of Bio- naïve patients (%)** | **Number of Bio-experienced patients (%)** |
| --- | --- | --- | --- |
| **Safety analysis set** | 341 (100.0) | 95 (100.0) | 246 (100.0) |
| **Completed patients** | 6 (1.8) | 4 (4.2) | 2 (0.8) |
| **Discontinued patients** | 59 (17.3) | 12 (12.6) | 47 (19.1) |
| **Suspended patients** | 2 (0.6) | 1(1.1) | 1 (0.4) |
| **Patients continuing treatment** | 274 (80.4) | 78 (82.1) | 196 (79.7) |

## Supplemental Table 2 Reasons for treatment discontinuation

| **Reasons for treatment discontinuation** | **Number of patients (% to discontinuation patients)** | **Number of patients  (% to discontinuation Bio-naïve patients)** | **Number of patients  (% to discontinuation Bio-experienced patients)** |
| --- | --- | --- | --- |
| **Number of discontinued patients** | 59 | 12 | 47 |
| **Patient choice** | 6 (10.2) | 1 (8.3) | 5 (10.6) |
| **Adverse event** | 8 (13.6) | 1 (8.3) | 7 (14.9) |
| **Lack of effectiveness** | 25 (42.4) | 6 (50.0) | 19 (40.4) |
| **Transfer hospital** | 12 (20.3) | 3 (25.0) | 9 (19.1) |
| **Not visit** | 3 (5.1) | 0 (0.0) | 3 (6.4) |
| **Other** | 5 (8.5) | 1 (8.3) | 4 (8.5) |

## Supplemental Table 3 Reasons for treatment suspension

| **Reasons for treatment suspension** | **Number of patients (% to suspension patients)** | **Number of patients  (% to suspension Bio- naïve patients)** | **Number of patients  (% to suspension Bio-experienced patients)** |
| --- | --- | --- | --- |
| **Number of suspended patients** | 2 | 1 | 1 |
| **Patient choice** | 1 (50.0) | 1 (100.0) | 0 (0.0) |
| **Adverse event** | 1 (50.0) | 0 (0.0) | 1 (100.0) |

## Supplemental Table 4 Administration interval

| **Visit** | | **Number of patients** |
| --- | --- | --- |
| **Change of administration interval** | **12 week** | 74 |
|  | **12 week → 8 week** | 207 |
|  | **8 week** | 20 |
|  | **8 week→ 12 week** | 1 |
|  | **More than once** | 39 |
